# Trehalose Maintains Bioactivity and Promotes Sustained Release of BMP-2 from Lyophilized CDHA Scaffolds for Enhanced Osteogenesis *In Vitro* and *In Vivo*

**DOI:** 10.1371/journal.pone.0054645

**Published:** 2013-01-24

**Authors:** Jun Zhao, Shaoyi Wang, Jianqiang Bao, Xiaojuan Sun, Xiaochen Zhang, Xiuli Zhang, Dongxia Ye, Jie Wei, Changsheng Liu, Xinquan Jiang, Gang Shen, Zhiyuan Zhang

**Affiliations:** 1 Department of Orthodontics, College of Stomatology, Ninth People’s Hospital, School of Medicine, Shanghai Jiao Tong University, Shanghai, China; 2 Department of Oral and Maxillofacial Surgery, College of Stomatology, Ninth People’s Hospital, School of Medicine, Shanghai Jiao Tong University, Shanghai, China; 3 Shanghai Research Institute of Stomatology, Shanghai Key Laboratory of Stomatology, Ninth People’s Hospital, School of Medicine, Shanghai Jiao Tong University, Shanghai, China; 4 Department of Physiology and Cell Biology, University of Nevada Reno, Reno, Nevada, United States of America; 5 Department of Oral and Maxillofacial Surgery, General Hospital of Ningxia Medical University, Yinchuan, Ningxia, China; 6 Key Laboratory for Ultrafine Materials of Ministry of Education, Engineering Research Center for Biomedical Materials of Ministry of Education, East China University of Science and Technology, Shanghai, China; National University of Ireland, Galway, Ireland

## Abstract

Calcium phosphate (Ca-P) scaffolds have been widely employed as a supportive matrix and delivery system for bone tissue engineering. Previous studies using osteoinductive growth factors loaded Ca-P scaffolds via passive adsorption often experience issues associated with easy inactivation and uncontrolled release. In present study, a new delivery system was fabricated using bone morphogenetic protein-2 (BMP-2) loaded calcium-deficient hydroxyapatite (CDHA) scaffold by lyophilization with addition of trehalose. The *in vitro* osteogenesis effects of this formulation were compared with lyophilized BMP-2/CDHA construct without trehalose and absorbed BMP-2/CDHA constructs with or without trehalose. The release characteristics and alkaline phosphatase (ALP) activity analyses showed that addition of trehalose could sufficiently protect BMP-2 bioactivity during lyophilization and achieve sustained BMP-2 release from lyophilized CDHA construct *in vitro* and *in vivo*. However, absorbed BMP-2/CDHA constructs with or without trehalose showed similar BMP-2 bioactivity and presented a burst release. Quantitative real-time PCR (RT-qPCR) and enzyme-linked immunosorbent assay (ELISA) demonstrated that lyophilized BMP-2/CDHA construct with trehalose (lyo-tre-BMP-2) promoted osteogenic differentiation of bone marrow stromal cells (bMSCs) significantly and this formulation could preserve over 70% protein bioactivity after 5 weeks storage at 25°C. Micro-computed tomography, histological and fluorescent labeling analyses further demonstrated that lyo-tre-BMP-2 formulation combined with bMSCs led to the most percentage of new bone volume (38.79% ±5.32%) and area (40.71% ±7.14%) as well as the most percentage of fluorochrome stained bone area (alizarin red S: 2.64% ±0.44%, calcein: 6.08% ±1.37%) and mineral apposition rate (4.13±0.62 µm/day) in critical-sized rat cranial defects healing. Biomechanical tests also indicated the maximum stiffness (118.17±15.02 Mpa) and load of fracture (144.67±16.13 N). These results lay a potential framework for future study by using trehalose to preserve growth factor bioactivity and optimize release profile of Ca-P based delivery system for enhanced bone regeneration.

## Introduction

Calcium phosphate (Ca-P) based synthetic bone substitutes are widely used in orthopaedic and maxillofacial surgeries [1], [2]. These materials have proved to be biocompatible and osteoconductive, but lack the osteogenic potential to promote bone healing of critical-sized defects [3]. In order to improve the osteogenic ability of the engineered bone grafts, osteoinductive growth factors are generally combined with the Ca-P biomaterials [4], [5]. Regeneration of bone defects can be accelerated by implanting a resorbable synthetic bone substitute that can locally deliver an appropriate amount of bioactive molecules at a desirable rate and concentration [6].

Bone morphogenetic proteins (BMPs), especially BMP-2, have shown an impressive ability in inducing orthotopic and ectopic new bone formation. Porous Ca-P scaffolds loaded with BMP-2 as grafting material has enabled enhanced bone regeneration in multiple animal models [7]–[11]. In most studies, BMP-2 was loaded onto the scaffolds as a diluted solution to construct delivery system for bone defects healing [9], [10], [12], [13]. However, this approach has several short-comings such as inaccurate dose, uncontrolled flow, and inconvenient handling, which can lead to issues such as wound complications, seroma formation, and bony resorption [14]. Furthermore, the implanted growth factors tend to be easily inactivated by heat, extreme pH, or proteases, resulting in unstable activity during the desired therapeutic period [15], . Increased concerns about these issues have led to the acceptance of a method for loading BMP-2 onto Ca-P scaffolds by coating it via lyophilization.

Lyophilization is an efficient approach to improving the stability of labile biopharmaceuticals [17]. Although this method has been commonly applied to overcome the instability and expand the shelf life of biopharmaceuticals, the lyophilization process generates both freezing and dehydration stresses, which could inactive proteins to various degrees [18], [19]. Thus, stabilizers are often used to protect proteins from lyophilization-derived stresses [20]. Saccharides are such nonspecific stabilizers that have been widely and successfully implemented to prevent biomolecules from denaturation by lyophilization process [18], [20]. Among different saccharides, disaccharides, such as trehalose and sucrose, have been extensively studied and generally recognized as some of the most effective stabilizers [21], [22]. Trehalose, a non-reducing disaccharide, is often used to stabilize the proteins as both cryo- and lyo-protectant and provides a glassy matrix by remaining amorphous during lyophilization [23], [24]. In comparison with other stabilizers, the excellent protective properties of trehalose have been ascribed to the high glass transition temperature, poor hygroscopicity, absence of reducing groups, and low crystallization rate [24]–[26]. Addition of trehalose to a growth factor delivery system associated with biomaterials and biomolecules will be highly valuable for preserving the bioactivity of loaded factors by lyophilization.

Recent studies employing organic materials such as absorbable collagen sponge for growth factors delivery lead to a large burst release immediately after implantation, which may arouse safety issues associated with administering high dosages of the drugs [27], [28]. Unlike these materials, a number of studies have demonstrated that lyophilized growth factors on Ca-P inorganic scaffolds exhibited an extremely slow release characteristic due to the high binding affinity between biomolecules and biomaterials [11], [29]–[31]. A common approach to optimize the release profile of bioactive growth factors from ceramics platforms is the addition of gelatin, albumin, or polylactic acid-based polymers. [32]–[36] However, these approaches could barely promote sustained release of the growth factors from Ca-P scaffolds effectively or might confront with a risk of the denaturation of the growth factors [37]. Nevertheless, some investigators found that adenovirus lyophilized in sucrose on hydroxyapatite showed a sustained release [38]. In addition, calcium phosphate scaffold coated with trehalose decreased the binding of basic fibroblast growth factor to the substrate and, in turn, facilitated the factor release from the scaffold [39]. Based on these findings, we hypothesize that lyophilization of growth factors in trehalose could be used as an alternative approach to developing a Ca-P based delivery system that could not only maintain the bioactivity of a protein, but also promote sustained release of growth factor for enhanced bone regeneration.

In the present study, a porous calcium-deficient hydroxyapatite (CDHA) scaffold was employed as the substrate for growth factor delivery. BMP-2 was loaded onto CDHA scaffold by lyophilization or physical adsorption following trehalose addition or no addition. The release characteristics of BMP-2/CDHA formulations and the bioactivities of BMP-2 releasates were determined *in vitro*. The effects of different formulations on osteogenic differentiation and proliferation of bone marrow stromal cells (bMSCs), as well as the preservation of the lyophilized formulation with addition of trehalose were also investigated. Thereafter, the release characteristics of BMP-2/CDHA formulations *in vivo* and BMP-2 delivery systems combined with bMSCs for bone regeneration were further evaluated in a critical-sized rat cranial defect model.

## Results

### *In vitro* Kinetics of BMP-2 Release from Scaffolds

**Figure 1:**
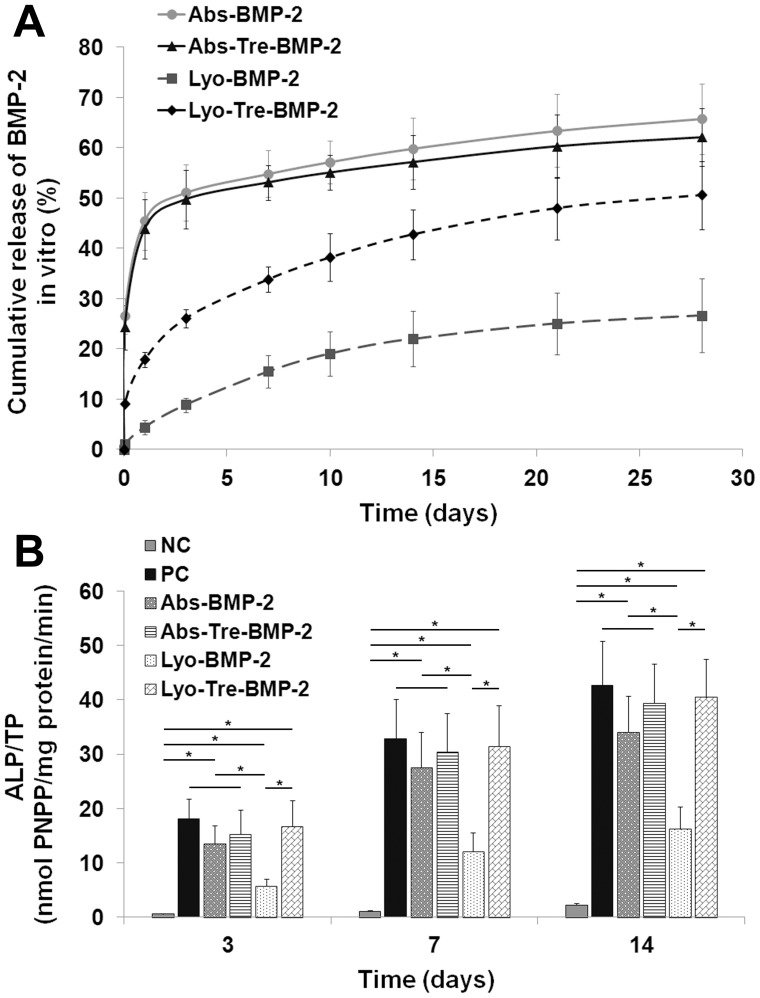
*In vitro* BMP-2 release profiles and ALP activity assay of BMP-2 releasates from different formulations. (A) *In vitro* release profiles of BMP-2 and (B) ALP activity assay of BMP-2 releasates from CDHA/BMP-2 delivery systems loaded by lyophilization or adsorption with or without addition of trehalose. Fresh BMP-2 solution (100 ng/ml) was used as a positive control (PC), and serum media containing no BMP-2 was used as the negative control (NC). Stars and lines indicate significance differences between groups at each time point (P<0.05).

The *in vitro* BMP-2 release profiles for different formulations were shown in Figure 1A. Both the lyophilized BMP-2 plus trehalose CDHA scaffold (lyo-tre-BMP-2) and lyophilized BMP-2 CDHA scaffold (lyo-BMP-2) showed a sustained release, but the overall release of lyo-tre-BMP-2 CDHA scaffold (50.59%, 3.04 µg) was significantly higher than that of lyo-BMP-2 CDHA scaffold (26.62%, 1.60 µg) (P<0.05). However, absorbed BMP-2 plus trehalose CDHA scaffold (abs-tre-BMP-2) and absorbed BMP-2 CDHA scaffold (abs-BMP-2) exhibited an initial burst, followed by a slow release over days 3 to 28. The cumulative releases of BMP-2 from abs-tre-BMP-2 (62.09%, 3.73 µg) and abs-BMP-2 (65.79%, 3.95 µg) CDHA scaffolds were statistically higher than those from lyo-tre-BMP-2 and lyo-BMP-2 CDHA scaffolds during the time frame (P<0.05). At each time point, the cumulative release of BMP-2 from lyo-tre-BMP-2 CDHA scaffold was significantly higher than that from lyo-BMP-2 CDHA scaffold (P<0.05). While, the cumulative release of BMP-2 from abs-tre-BMP-2 CDHA scaffold was similar with that from the abs-BMP-2 CDHA scaffold.

### BMP-2 Bioactivity Released from Scaffolds

The bioactivities of released BMP-2 were shown in Figure 1B. The same amount of BMP-2 releasates from four BMP-2/CDHA formulations and fresh BMP-2 significantly enhanced ALP activity at days 3, 7 and 14, when compared to the negative control (P<0.05). However, the bioactivity of BMP-2 releasate from lyo-BMP-2 CDHA scaffold was statistically lower than that from other three scaffolds and positive control group (P<0.05). There was no significant difference among the releasates from lyo-tre-BMP-2, abs-BMP-2, abs-tre-BMP-2 CDHA scaffolds and the fresh BMP-2 in terms of ALP activity at each time point.

### Effect of Scaffolds on bMSCs Proliferation

**Figure 2:**
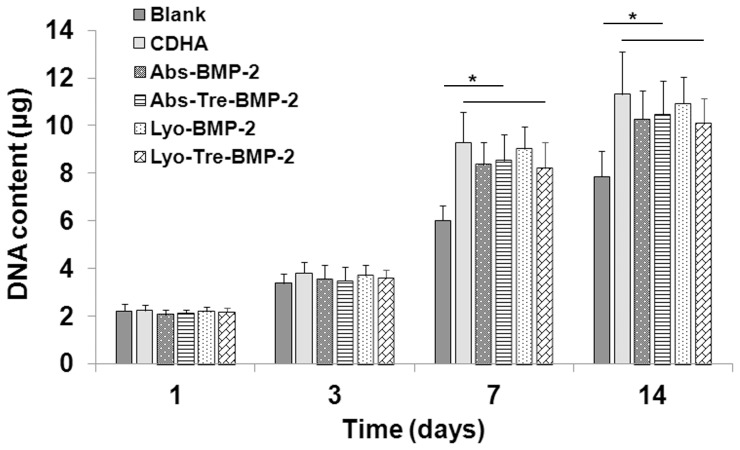
Effects of different formulations on bMSCs proliferation. DNA assay for proliferation of bMSCs cocultured with various CDHA/BMP-2 delivery systems at days 1, 3, 7 and 14. Stars and lines indicate significance differences between groups at each time point (P<0.05).

Proliferation of bMSCs cocultured with various scaffolds was determined by DNA assay, as shown in Figure 2. Proliferation of cells with CDHA scaffold, lyo-tre-BMP-2, lyo-BMP-2, abs-tre-BMP-2 and abs-BMP-2 CDHA scaffolds were almost at the same level. After culturing for 7 and 14 days, cell proliferation with these five scaffolds was significantly higher than that of control cells without any scaffold (P<0.05).

### Effect of Scaffolds on bMSCs Osteogenic Differentiation

**Figure 3:**
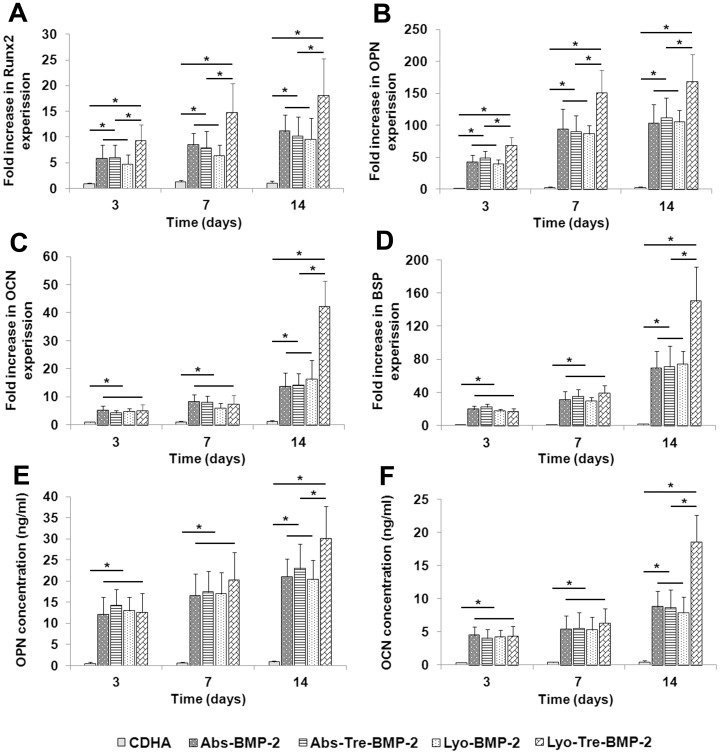
Effects of different formulations on bMSCs osteogenic differentiation. RT-qPCR analysis of gene expression of osteogenic markers (A) runx2 (Runx2), (B) osteopontin (OPN), (C) osteocalcin (OCN) and (D) bone sialoprotein (BSP) for bMSCs cocultured with various CDHA/BMP-2 delivery systems and CDHA alone scaffold relative to the expression of that for bMSCs with no grafts at days 3, 7 and 14, all values normalized to GAPDH. Protein levels for (E) OPN and (F) OCN in cell culture supernatant for bMSCs cocultured with different CDHA/BMP-2 delivery systems and CDHA alone scaffold. Stars and lines indicate significance differences between groups at each time point (P<0.05).

Osteogenic differentiation of bMSCs cocultured with BMP-2/CDHA scaffolds was determined by quantitative real-time PCR (RT-qPCR) (Figure 3). For the lyo-tre-BMP-2 group, the mRNA expression of Run×2 showed initial upregulation at day 3 and then increased continuously from days 7 to 14 (Figure 3A); OPN expression increased dramatically from day 3 to 7 and then slightly enhanced from day 7 to 14 (Figure 3B); OCN and BSP expression showed a slight rise which was followed by a remarkable increase between day 7 and 14 (Figure 3C, D). For lyo-BMP-2, abs-tre-BMP-2 and abs-BMP-2 groups, the mRNA expressions of Runx2, OPN, OCN and BSP resembled those of lyo-tre-BMP-2 group, but the increase folds were significantly lower than those of lyo-tre-BMP-2 group at day 14 (P<0.05). The mRNA expressions of Runx2 and OPN for these three groups at day 3 and 7 were also significantly lower than that for lyo-tre-BMP-2 group (P<0.05). No statistical difference of mRNA expression for the transcripts of osteogenic markers was observed among these three groups at each time point. Overall, the mRNA expression of osteogenic markers in the CDHA alone group was statistically lower than that of the other four groups at each time point (P<0.05).

OPN and OCN protein levels for bMSCs cocultured with BMP-2/CDHA scaffolds were further determined by enzyme-linked immunosorbent assay (ELISA) (Figure 3E, F). After 14 days culture, the OPN and OCN protein levels for lyo-tre-BMP-2 group were significantly increased than those for other four groups (P<0.05). The OPN and OCN protein levels for CDHA alone group were statistically lower than those for BMP-2 loaded groups at each time point (P<0.05). No statistical difference of protein contents for OPN and OCN was observed among lyo-BMP-2, abs-tre-BMP-2 and abs-BMP-2 groups at each time point.

### Stability of Lyophilized BMP-2 on CDHA Scaffold with Trehalose

**Figure 4:**
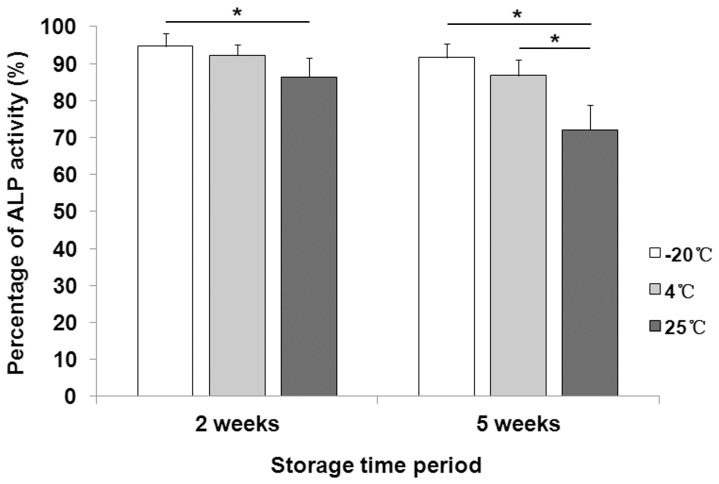
Stability of lyophilized BMP-2 on CDHA scaffold with trehalose. Evaluation of the percentage of BMP-2 bioactivity of lyophilized CDHA/BMP-2 with trehalose under different temperature conditions: −20°C, 4°C, and 25°C. After 2 and 5 weeks storage, 100 ng/ml of BMP-2 releasate was collected for ALP activity assay. The same amount of fresh BMP-2 was compared as standard for ALP assay to determine the change of protein bioactivity. Stars and lines indicate significance differences between groups at each time point (P<0.05).

The effects of different storage temperatures and periods prior to implantation on the bioactivity of lyophilized BMP-2 on CDHA scaffold with trehalose are presented in Figure 4. Compared with fresh BMP-2, over 90% of bioactivity was retained after 2 weeks storage at −20°C and 4°C, and 5 weeks storage at −20°C. About 85% of bioactivity was remained after 2 weeks storage at 25°C, and 5 weeks storage at 4°C. BMP-2 bioactivity decreased to about 70% after 5 weeks storage at 25°C. The BMP-2 bioactivity of the lyo-tre-BMP-2 formulation stored at 25°C after 2 weeks was significantly lower than that stored at −20°C during the same time frame (P<0.05). The BMP-2 bioactivity of this formulation at 25°C after 5 weeks was significantly lower than that at −20°C and 4°C after the same storage time period (P<0.05).

### Scanning Electron Microscopy (SEM)

The surface morphology of scaffolds and the interactions of bMSCs within the scaffolds were evaluated by SEM (Figure S1). After 24 hours combination, cells were fully spreading and growing (Figure S1, A2–D2). Nominal differences in cellular adhesion were observed among each group. At days 7 after cell seeding, the pores of lyo-tre-BMP-2 CDHA scaffold were deposited with abundant, dense extracellular matrix associated with cell layers (Figure S1, A3). Visually, less extracellular matrix formed on the lyo-BMP-2 and abs-BMP-2 CDHA scaffolds (Figure S1, B3, C3) than that on the lyo-tre-BMP-2 scaffolds. Only a thin cell layer grew on the surface of CDHA scaffold (Figure S1, D3).

### General Observation

After surgery, the rats were kept in the clean conditions with adequate water and food. All the animals recovered well. Slight post-surgical edema was observed at the recipient site in each rat. This disappeared 3–5 days after the procedure. There was no sign of infection at any time. The average weight of the rats before sacrifice showed a slight increase of 30 g±10 g above the initial average weight.

### *In vivo* Kinetics of BMP-2 Release from Scaffolds

Based on the above results, we found that the addition of trehalose did not affect the release profile and bioactivity of BMP-2 from the scaffolds when the BMP-2 was loaded by physical adsorption. Meanwhile, the trehalose had no stimulative or suppressive effect on osteogenic differentiation and proliferation of bMSCs with BMP-2. Thus, the abs-tre-BMP-2 group was not involved in the following experiment.

**Figure 5:**
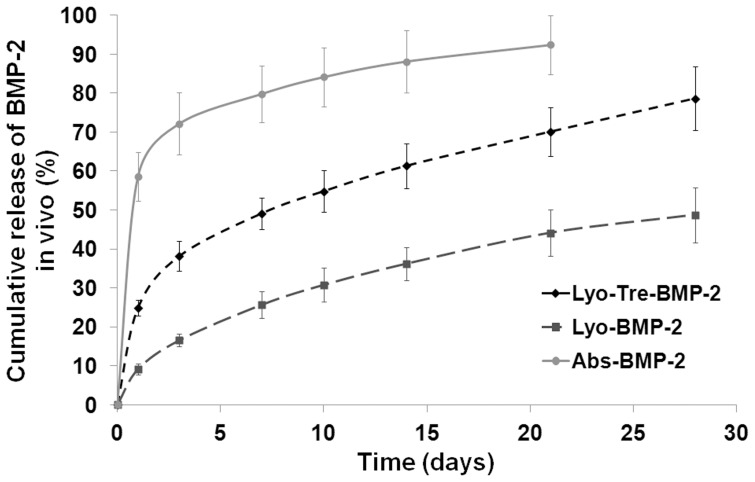
*In vivo* BMP-2 release profiles of different formulations. *In vivo* release profiles of BMP-2 from lyophilized CDHA/BMP-2 with or without trehalose and adsorbed CDHA/BMP-2 formulations after implantation in a rat calvarial bone defect.

The remaining ^125^I-BMP-2 in the absorbed and lyophilized formulations was measured during weeks 3 and 4, respectively (Figure 5), due to the fact that no radioactivity could be detected for abs-BMP-2 formulation at 4 weeks time point. The abs-BMP-2 formulation showed a burst release of approximately 60% protein within the first day. At the end of the third week a total of over 90% of loaded BMP-2 had been released. Unlikely, both of the lyophilized formulations showed a sustained release, but the cumulative release of BMP-2 from lyo-tre-BMP-2 formulation was significantly higher than that from lyo-BMP-2 formulation at each time point (P<0.05). The lyo-tre-BMP-2 formulation released about 25% of loaded BMP-2 within the first day, while only 10% was released from the lyo-BMP-2 formulation. At the end of the fourth week, a total of about 80% of loaded BMP-2 had been released from the lyo-tre-BMP-2 formulation, which was significantly higher than the 50% released from the lyo-BMP-2 formulation (P<0.05).

### Micro-computed Tomography (μ-CT) Measurement

**Figure 6:**
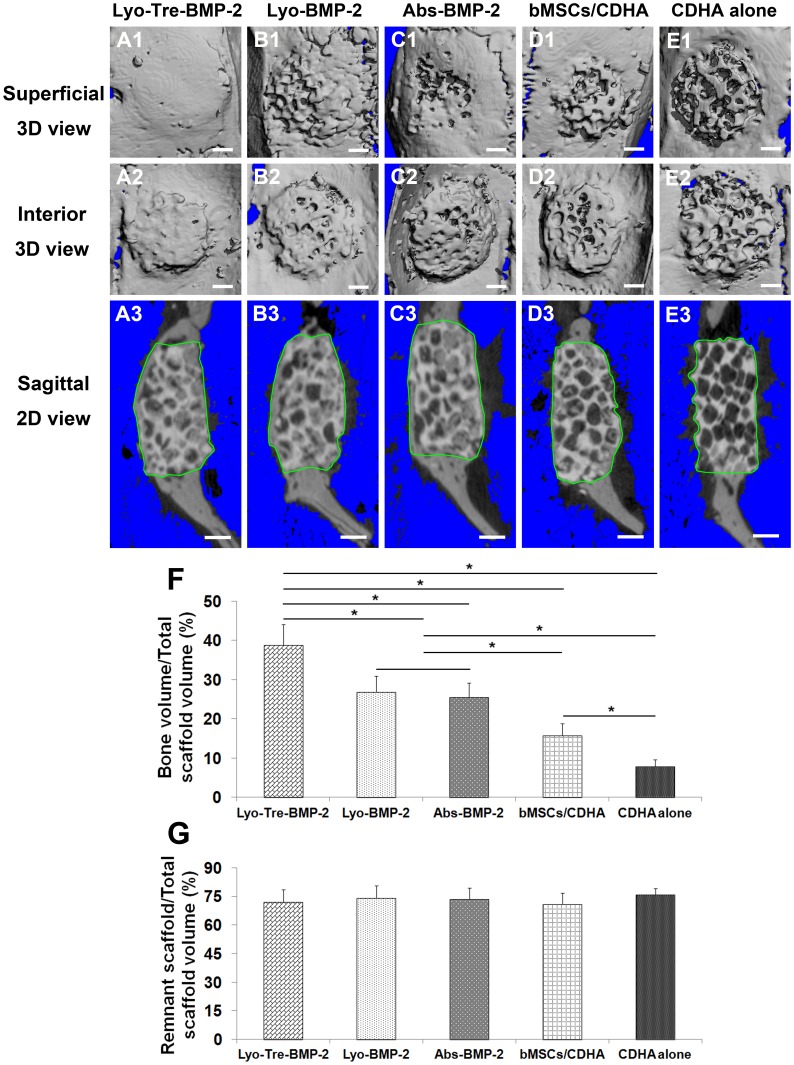
μ-CT evaluation and morphometric analysis of calvarial bone repair. Representative 3D and 2D μ-CT images of calvarial bone defects taken 5 weeks after implantation. Defects were treated with (A1–A3) lyo-tre-BMP-2, (B1–B3) lyo-BMP-2, (C1–C3) abs-BMP-2, (D1–D3) CDHA scaffold combined with bMSCs, and (E1–E3) CDHA scaffold alone. The boundaries of the volume of interest were outlined (green) on the longitudinal 2D tomograms. 3D images: A1–E1 (superficial view) and A2–E2 (interior view). 2D images: A3–E3 (longitudinal view). Scale bar = 1 mm. Morphometric analysis of (F) bone volume/total scaffold volume and (G) remnant scaffold/total scaffold volume by μ-CT for each group at 5 weeks post-operation. Stars and lines indicate significance differences between groups (P<0.05).

3D morphology and 2D original slices of the repaired calvarial bone were presented in Figure 6A–E. New bone formation was substantial in the calvarial bone defects after 5 weeks for lyo-tre-BMP-2 group, with induced bone volume on the bilateral sides of the calvaria that were equivalent to the cortical level on pseudo-3D displays and almost completely filled scaffold pores on 2D slices (Figure 6A1–A3). Bone formation was relatively less for lyo-BMP-2 (Figure 6B1–B3) and abs-BMP-2 group (Figure 6C1–C3) compared with lyo-tre-BMP-2 group. No significant difference for bone healing among lyo-BMP-2, abs-BMP-2 and bMSCs/CDHA groups was found from 3D observation, but advanced new bone formed inside of the scaffold pores for lyo-BMP-2 and abs-BMP-2 group than that for bMSCs/CDHA group (Figure 6D1–D3) from 2D observation. Minimal bone formation was observed in CDHA alone group (Figure 6E1–E3).

The percentages of new bone volume and remnant scaffold volume in the defect site among the mean scaffold volume were calculated by morphometrical analysis. The percentage of bone volume was higher in lyo-tre-BMP-2 group than other four groups (P<0.05) (Figure 6F). There was no significant difference between lyo-BMP-2 and abs-BMP-2 groups. Percentage of bone volume for bMSCs/CDHA group was statistically lower than that for lyo-BMP-2 and abs-BMP-2 groups (P<0.05), but significantly higher than that for CDHA alone group (P<0.05). No statistical difference for the percentage of remnant scaffold volume was found among five groups (Figure 6G).

### Fluorochrome Labeling Histomorphometrical Analysis

**Figure 7:**
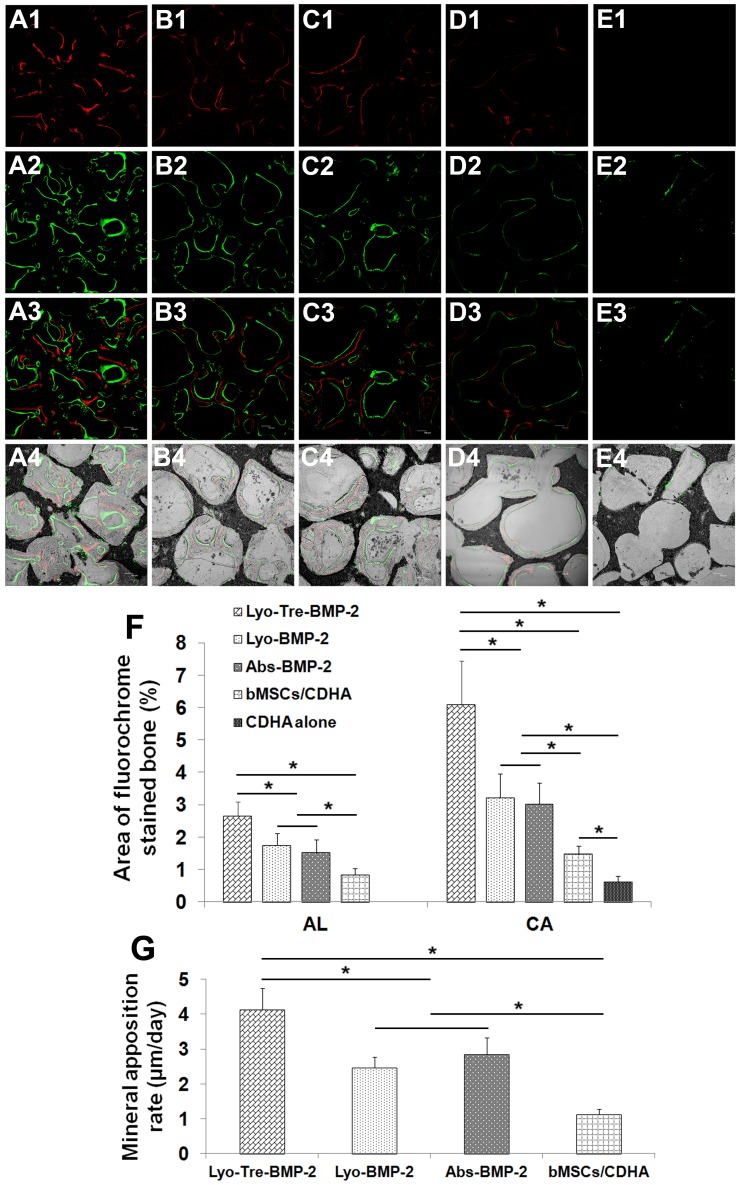
Fluorescent labelings and histomorphometric analysis for percentage of fluorochrome area and mineral apposition rate. New bone formation and mineralization was determined by CA and AL fluorescent labelings, which represented the mineralization level at 2 (A1–E1) and 4 (A2–E2) weeks after operation. A3–E3 represented merged images of the fluorochromes. A4–E4 represented the merged images of the fluorochromes together with plain CLSM image. A1–A4 represented lyo-tre-BMP-2 group (50×); B1–B4 represented lyo-BMP-2 group (50×); C1–C4 represented abs-BMP-2 group (50×); D1–D4 represented bMSCs/CDHA group (50×); E1–E4 represented CDHA scaffold alone group (50×). Histomorphometric analysis of (F) the percentage of each fluorochrome area and (G) mineral apposition rate for each group. Stars and lines indicate significance differences between groups (P<0.05).

Fluorochrome labels were administered at successive intervals of 2 and 4 weeks post-operation to evaluate new bone formation and mineralization (Figure 7). The percentage of alizarin red S (AL) labeling (red) at 2 weeks for lyo-tre-BMP-2 group was significantly higher than that for other three groups (P<0.05). No significant difference was found for AL labeling between lyo-BMP-2 and abs-BMP-2 groups. The percentage of AL labeling for bMSCs/CDHA group was statistically lower than that for lyo-BMP-2 and abs-BMP-2 groups (P<0.05). The AL labeling for CDHA alone group could not be detected at this early stage. At 4 weeks, calcein (CA) labeling for CDHA alone group could be detected but the percentage for CA labeling was significantly lower than that for other four groups (P<0.05). The tendency of CA labeling for other groups at 4 weeks was similar to that of AL labeling at 2 weeks (Figure 7F).

At 2–4 weeks post-operation, lyo-tre-BMP-2 group presented the highest mineral apposition rate among four groups (P<0.05) (Figure 7G). The mineral apposition rates for lyo-BMP-2 and abs-BMP-2 groups were at the same level, which were significantly higher than that for bMSCs/CDHA group (P<0.05). Due to the AL labeling for the CDHA alone group could not be detected, we failed to present the mineral apposition rate for this group during this time period.

### Histological and Histomorphometrical Analysis of Bone Regeneration

**Figure 8:**
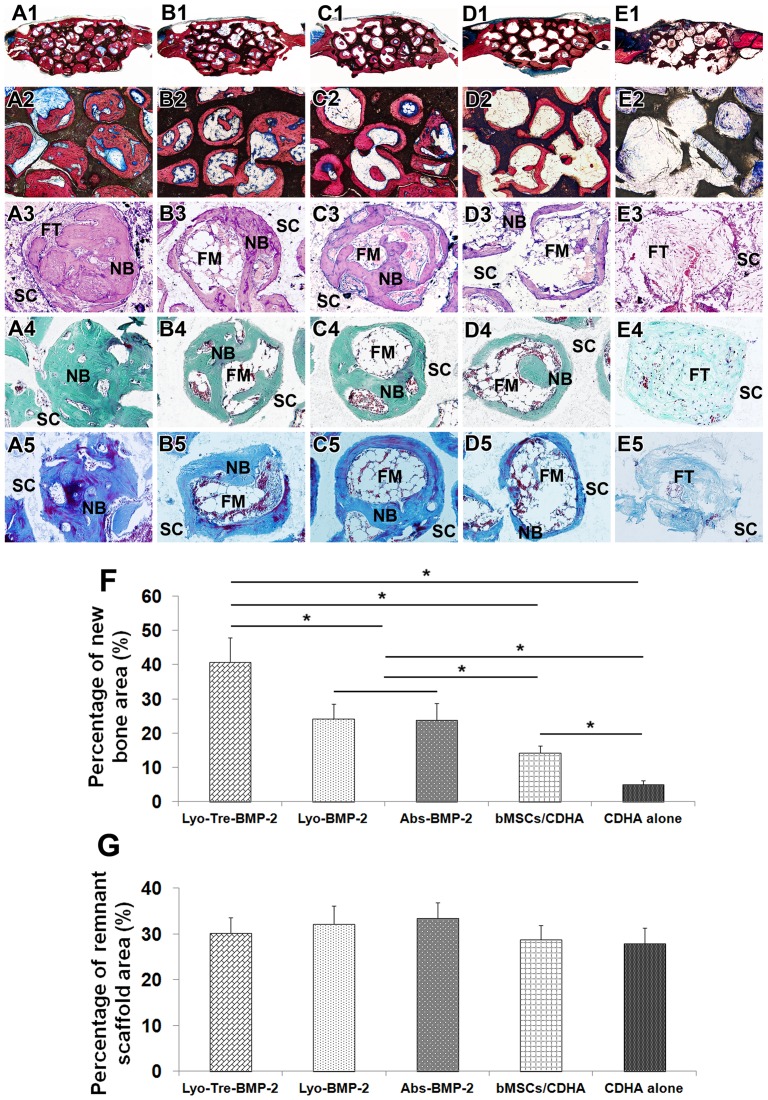
Histology and histomorphometry analysis of calvarial bone repair 5 weeks post-operation. The photomicrograph of new bone formation in repaired calvarial bone defects from undecalcified (A1–E1, A2–E2) and decalcified samples (A3–E3, A4–E4, A5–E5). The whole images of the representative slices for each group: lyo-tre-BMP-2 (A1, 12.5×), lyo-BMP-2 (B1, 12.5×), abs-BMP-2 (C1, 12.5×), bMSCs/CDHA (D1, 12.5×), and CDHA scaffold alone (E1, 12.5×) group. A2–E2 (100×) represent the high magnitude images from A1–E1 respectively. Bone tissue staining consists of HE (A3–E3, 200×), Goldner’s Trichrome (A4–E4, 200×; green = bone, red = osteoid, purple = cartilage), and Masson’s Trichrome (A5–E5, 200×; dark blue = bone, red = cortical bone). From undecalcified sections stained with Van Gieson’s picro fuchsin, substantial bone formation in lyo-tre-BMP-2 group, less bone formation in lyo-BMP-2 and abs-BMP-2 groups, only a thin layer bone formation along the pore walls in bMSCs/CDHA group and no obvious bone formation in CDHA alone group were found. From decalcified sections stained by HE, substantial new bone formed with little fibrous connective tissue infiltration in the pores in lyo-tre-BMP-2 group. Less newly formed bone was observed along the wall of pores with small amount of fatty marrow tissue interspersed in lyo-BMP-2 and abs-BMP-2 groups. Only a little newly formed bone with large amount of fatty marrow tissue was found in bMSCs/CDHA group. Defects filled with CDHA scaffolds alone were found with a large quantity of fibrous connective tissue in the area of defects with visible blood vessels close to the surface of the scaffold. Decalcified sections stained by Goldner’s trichrome showed more mineralized bone matrix in lyo-tre-BMP-2 group than other groups. Decalcified sections stained by Masson’s trichrome showed more matured collagen in the bone tissue of lyo-tre-BMP-2 group than other four groups. FM: fat marrow; FT: fibrous tissue; NB: new bone; SC: scaffold. Histomorphometrical analysis for the area of (F) new bone formation and (G) remnant scaffold by undecalcified sections stained with Van Gieson’s picro fuchsin for each group at 5 weeks post-operation. Stars and lines indicate significant differences between groups (P<0.05).

Histological evidence further supported the above findings (Figure 8). From undecalcified sections stained by Van Gieson’s picro fuchsin, substantial bone formation in lyo-tre-BMP-2 group (Figure 8A1, A2), less bone formation in lyo-BMP-2 (Figure 8B1, B2) and abs-BMP-2 (Figure 8C1, C2) groups, only a thin layer bone formation along the pore walls in bMSCs/CDHA group (Figure 8D1, D2) and no obvious bone formation in CDHA alone group (Figure 8E1, E2) were found. The percentage of new bone area in lyo-tre-BMP-2 group was statistically higher than that for other four groups (P<0.05) (Figure 8F). There was no significant difference for new bone percentage between lyo-BMP-2 and abs-BMP-2 groups. New bone percentage for bMSCs/CDHA group was significantly lower than that for lyo-BMP-2 and abs-BMP-2 groups (P<0.05), but still higher than CDHA alone group (P<0.05). The percentage of remnant scaffold area was at the same level for each group (Figure 8G).

From decalcified sections stained by hematoxylin-eosin (HE), substantial new bone formed with little fibrous connective tissue infiltration in the pores in lyo-tre-BMP-2 group (Figure 8A3). Less newly formed bone was observed along the wall of pores with small amount of fatty marrow tissue interspersed in the lyo-BMP-2 (Figure 8B3) and abs-BMP-2 (Figure 8C3) groups. Only a small amount of newly formed bone with large amount of fatty marrow tissue was found in bMSCs/CDHA group (Figure 8D3). Defects filled with CDHA scaffolds alone were found with a large quantity of fibrous connective tissue in the area of defects with visible blood vessels close to the surface of the scaffold (Figure 8E3). Decalcified sections stained by Goldner’s trichrome (Figure 8A4–E4) showed more mineralized bone matrix in lyo-tre-BMP-2 group than other groups. Decalcified sections stained by Masson’s trichrome (Figure 8A5–E5) showed more matured collagen in the bone tissue of lyo-tre-BMP-2 group than other four groups.

### Biomechanical Analysis

**Figure 9:**
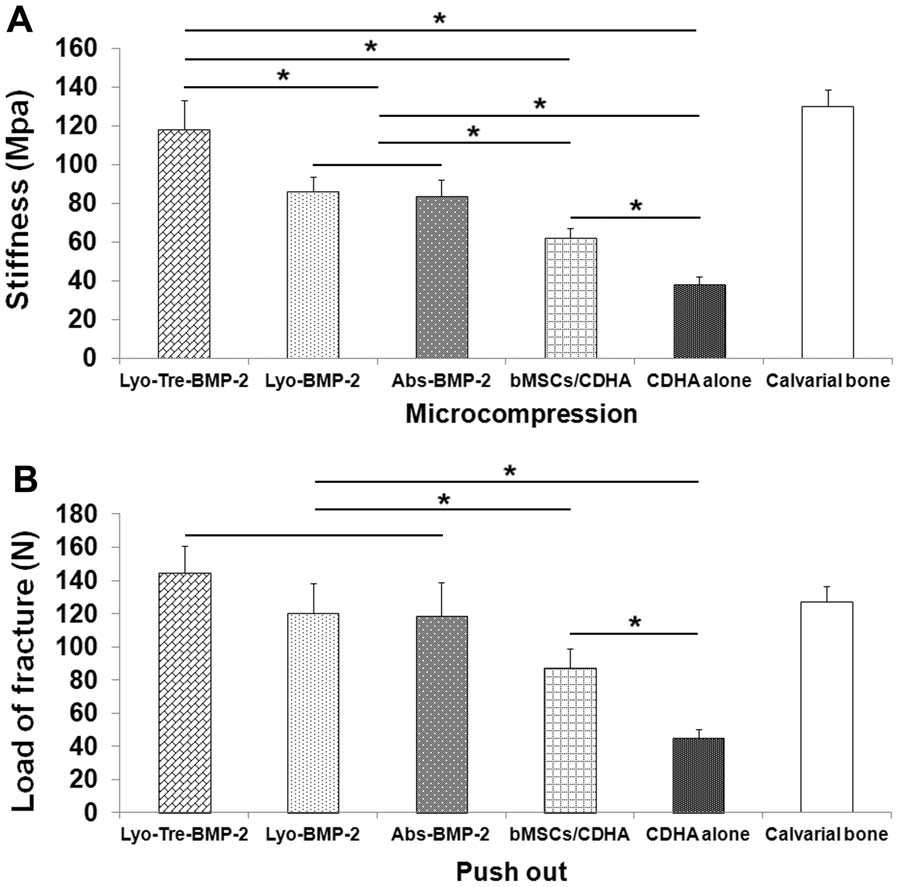
Biomechanical analysis of repaired calvarial bone defects. (A) Micro-compression test and (B) push-out test were performed at 5 weeks after operation. Regenerated tissue within lyo-tre-BMP-2 group exhibited comparable stiffness and push-out strength with intact bone. Stars and lines indicate significant differences between groups (P<0.05).

To evaluate the biomechanical properties of repaired calvarial defects, micro-compression and push-out tests were performed on harvested calvaria defects of each specimen. Lyo-tre-BMP-2 group showed similar stiffness with the normal calvarial bone, which was statistically higher than other four groups (P<0.05). Lyo-BMP-2 group possessed a similar compressive strength with abs-BMP-2 group. The compressive strength for bMSCs/CDHA group was significantly lower than that for lyo-BMP-2 and abs-BMP-2 groups (P<0.05), but statistically higher than that for CDHA alone group (P<0.05) (Figure 9A). The push-out test was conducted to evaluate the functional mechanical integration of the constructs with host calvaria (Figure 9B). At 5 weeks post-operation, no significant difference was found for the load of fracture among lyo-tre-BMP-2, lyo-BMP-2, abs-BMP-2 and normal calvarial bone groups. The load of fracture for bMSCs/CDHA group was significantly lower than that for these four groups (P<0.05), but higher than that for CDHA alone group (P<0.05).

## Discussion

Calcium phosphate biomaterials have been considered to be suitable candidates for BMP-2 delivery, due to their space-providing and biodegradable properties [1]–[5], [14], [29]. BMP-2 release profile of the Ca-P carrier was variable depending on the loading method of the factor and the morphology of the substrate. In prior studies, when BMP-2 was loaded onto Ca-P scaffold by passive adsorption, the composite showed a burst release [40], which was dramatically distinguishable from lyophilized BMP-2 scaffold that presented a very slow or decreased release characteristic [11], [41]. The similar observation was found in present study as well. This discrepancy between lyophilized and adsorbed BMP-2 Ca-P scaffolds might be attributed to the high binding affinity of BMP-2 with Ca-P materials generated during the lyophilization process [11], [41]. Meanwhile, using the same amount of BMP-2 releasate to examine the bioactivity of BMP-2 loaded by this two loading methods, we found that the ALP expression of lyophilized BMP-2 was lower than that of fresh BMP-2 which indicated the osteoinductive potential of BMP-2 might be partially subtracted by lyophilization. During the freezing and dehydration process, some factors might undergo irreversible structural changes which cause the protein inactivation [17], [42]. In order to prevent proteins from denaturation, a variety of strategies have been applied to preserve protein activity during lyophilization which typically involve the use of saccharides. Trehalose, a natural disaccharide present in diverse organisms, widely protects biostructures such as proteins and membranes from damage due to dehydration, heat, or freeze as a nonspecific process [21]–[26], [43]. In the present study, when trehalose was added into BMP-2 solution before lyophilization, the bioactivity of BMP-2 was preserved as a fresh agent (Figure 1B). Although adsorbed BMP-2 maintained the bioactivity as fresh BMP-2 as well, the osteogenic differentiation of bMSCs cocultured with a abs-tre-BMP-2 CDHA scaffold was significantly lower than that of a lyo-tre-BMP-2 CDHA scaffold (Figure 2). This result suggested that the BMP-2 release profile of delivery devices by different loading methods play a vital role in promoting osteogenic differentiation. Sustained release of BMP-2 is more effective than a bolus release for osteogenesis of bMSCs.

Meanwhile, addition of trehalose significantly enhanced BMP-2 release from a CDHA scaffold in lyophilized groups. Trehalose dripped on a CDHA surface might inhibit the binding of BMP-2 to Ca-P surface during lyophilization, thereby enabling increased BMP-2 release [39]. On the other hand, we found that addition of trehalose in absorbed BMP-2/CDHA scaffold barely altered the release profile and bioactivity of BMP-2, as well as osteogenic differentiation of bMSCs. This result suggested that trehalose would not enhance osteogenic differentiation of bMSCs with BMP-2. Based on the above findings, it would be reasonable to interpret the promotion of osteogenic differentiation for bMSCs cocultured with lyo-tre-BMP-2 CDHA scaffold compared with that for lyo-BMP-2 CDHA scaffold, which could be attributed to substantial bioactivity preservation and enhanced BMP-2 release.

With regard to the proliferation of bMSCs cocultured with various delivery systems, DNA assays showed no significant difference among the BMP-2 loaded CDHA and CDHA alone groups. However, the proliferation of these groups was significantly higher than that in the control group without any scaffold after 7 and 14 days culture. This result might be due to the main constituents of the scaffold which could induce bMSCs proliferation but not BMP-2 [44], [45]. With trehalose in the scaffold, the DNA amount of bMSCs was similar with that of BMP-2 loaded CDHA scaffolds without trehalose and CDHA alone scaffold, which indicated that trehalose had no obvious promotive or inhibitory effect on bMSCs proliferation.

The *in vivo* release kinetics of BMP-2 from the CDHA scaffolds resembled the *in vitro* kinetics independent of the administration strategies. Similar observation was found in other ectopic and orthotopic animal models as well [46]–[48]. However, compared with the cumulative release of loaded BMP-2 *in vitro*, the overall *in vivo* release was even higher during the same time frame. The accelerated degradation in the more protein-rich *in vivo* environment might have contributed to the underestimation of the cumulative release *in vitro* [49]. Apart from *in vitro* and *in vivo* hydrolyses of the calcium phosphate in an aqueous environment, cellular enzymatic actions could accelerate implant degradation upon implantation. The abundant blood circulation in craniofacial region also could promote the cellular metabolic process, which in turn facilitated implant degradation *in vivo* [50].

To determine the *in vivo* bone formation ability, various scaffolds combined with bMSCs and CDHA scaffold alone were prepared and implanted to the rat cranial defects. CDHA scaffolds by themselves were able to induce invading reparative cells from the surrounding host tissue to repair the defects, but strictly limited around the periphery of the defects with no bridging observed. Through step-wise fluorescent labeling, no fluorescence AL was detected at two weeks post operation, indicating that the nature new bone deposition and mineralization failed to occur at this early time point. Previous study found osteoblast migrated out of autogenous bone fragments and reached confluence in 9–16 days [51]. Therefore, the new bone matrix formation and mineralization might take place beyond this time frame. A small increase in the amount of new bone formation inside of the scaffold pores was found from μ-CT and histological analyses for defects filled with bMSCs/CDHA composites. Fluorescent labeling analysis also demonstrated that new bone deposition and mineralization occurred at 2 weeks post operation. These results suggested that implanted bMSCs contributed to the new bone formation. The *in vivo* osteogenesis for abs-BMP-2, lyo-BMP-2 and lyo-tre-BMP-2 groups was anticipated due to the results from *in vitro* studies. Lyo-tre-BMP-2 CDHA scaffold seeded with bMSCs resulted in promoted new bone formation and mineralization, as well as improved biomechanical properties of the newly formed bone. Sustained release of bioactive BMP-2 promoted osteogenic differentiation of implanted bMSCs and invading reparative cells for enhanced bone regeneration in the defect site. However, the push-out strengthes, which indicating mechanical integration of the implant with the host bone, was similar for abs-BMP-2, lyo-BMP-2 and lyo-tre-BMP-2 groups. This result could be explained by the extensive peripheral healing of bone occurring within BMP-2 treated defects. As was demonstrated in other cranial defect model [52], the push-out strength for abs-BMP-2 and lyo-BMP-2 groups might be lower than that for lyo-tre-BMP-2 group at early time point, but caught up with lyo-tre-BMP-2 group to the same level at the end of observation time point.

As for the degradation of CDHA scaffolds, no difference was found in the percentage of remnant CDHA scaffold volume and area among the different groups from μ-CT and histological analyses. This observation was similar with our previous study in which a lyophilized BMP-2 CDHA scaffold with sulfated chitosan coating (CDHA/BMP-2/SCS) was used in the same rat model for 8 weeks [31]. The remnant CDHA scaffolds for CDHA/BMP-2/SCS, CDHA/BMP-2 (lyophilized BMP-2 loaded CDHA scaffold without sulfated chitosan coating) and CDHA alone groups were also at the same level at 8 weeks post-operation. In other researchers’ studies by using CDHA scaffolds in a rabbit radius defect model, the degradation of CDHA scaffolds was increased by the addition of mesenchymal stem cells and/or growth factors after 16 weeks implantation *in vivo* [53], [54]. Similar observation was also found in a goat maxillary sinus augmentation model at 3 months post operation [55]. Additional mesenchymal stem cells or (and) growth factors, which could recruit progenitor cells or osteoclasts, might have a positive effect on the degradation of CDHA scaffolds and bone remolding. Nevertheless, no statistical difference of remaining CDHA among the groups was found in our study, which was probably due to the different animal model and observation time point. In future studies, the degradation of CDHA scaffold in the rat model should be evaluated for longer period.

As mentioned above, gelatin, albumin, or polylactic acid-based polymers were added with Ca-P based delivery system to optimize the release profile of growth factors. Studies employing BMP-2/gelatin/CPC system reported that the overall BMP-2 release ranged from 25% to 40% *in vitro* at 28 days observation time point [32], [40]. Researchers found that BMP-2 loaded porous CPC scaffold pretreated with albumin could promote BMP-2 release as well [33]. This delivery system presented about 20% release *in vitro* and 80% release when implanted subcutaneously in the back of rats after 28 days. Other researchers also employed polylactic acid-based polymers such as poly(lactic-co-glycolic acid) (PLGA) to modify Ca-P based delivery system. The overall BMP-2 release by this approach varied from 30% to 80% *in vivo* at 28 days, depending on different animal models [36], [48]. In the same rat cranial defect model, the overall BMP-2 release by BMP-2/PLGA/CPC was approximately 30% *in vivo* and the percentage of newly formed bone area was only 16% at 12 weeks post operation [36]. While, lyo-tre-BMP-2 CDHA formulation presented nearly 50% *in vitro* release and 80% *in vivo* release in present study. In comparison with previous studies, lyophilized BMP-2 with trehalose on CDHA scaffolds promoted sustained release of growth factors from Ca-P substrates more effectively both *in vitro* and *in vivo*.

Organic bone graft substitutes such as BMP-loaded collagen sponge, are commonly utilized in bone regeneration in different animal models and multiple clinical settings. However, collagen sponge and other organic material based delivery systems usually lead to a large burst release of growth factors immediately after implantation (within 7 days) and may actually cause some untoward effects associated with administering high dosages of the drugs, such as wound complications, seroma formation, and bony resorption [27], [28]. On the contrary, Ca-P carriers present a prolonged retention of these factors and perform better to avoid some of these problems [33]. In this study, addition of trehalose optimized the release profile of BMP-2 from lyophilized CDHA scaffold without any untoward effects and promoted sufficient new bone formation *in vivo*. Therefore, the lyophilized BMP-2 CDHA scaffold with trehalose holds an advantage over BMP-loaded collagen sponge and other organic material based delivery systems for the property of controlled release of growth factor for bone regeneration.

Furthermore, few studies assessed the bioactivity of growth factors loaded on the delivery system for bone regeneration, which could be easily inactivated by storage temperature and period. Le Nihouannen et al. reported the bioactivity of absorbed factors on a brushite cement scaffold by addition of trehalose. In their study, about 60% of the bioactivity of loaded factors was retained after 5 weeks storage at room temperature. However, the release profile of loaded factors was unexplored in the study [6]. In our study, addition of trehalose in lyophilization formulation could preserve BMP-2 bioactivity during scaffold preparation process and maintain over 70% of the bioactivity of BMP-2 for at least 5 weeks at 25°C. By preserving the stability and controlling the release of bioactive growth factors for synthetic bone graft substitutes, new delivery systems will become of a high interest to regenerate damaged bone tissue for clinic applications.

In a previous study, we fabricated a lyophilized BMP-2 CDHA delivery system with sulfated chitosan coating (CDHA/BMP-2/SCS) [31]. This composite presented an enhanced and sustained release profile of BMP-2. The ionic interaction between negatively charged polysaccharide chains in sulfated chitosan molecules and positively charged cavities in BMP-2 molecules increased the affinity between sulfated chitosan and BMP-2. This ionic interaction was responsible for the improved BMP-2 release from lyophilized CDHA scaffolds. The mechanism for accelerating BMP-2 release was different from that by using trehalose in this study. Researchers reported that there were three types of functional groups (-OH, -NH_2_, and -COO^−^) through which BMP-2 could interact with hydroxyapatite crystallite, and that the coulomb forces involved in these interactions might affect the release of BMPs from the Ca-P materials [56]. Addition of trehalose in BMP-2 solution before lyophilization not only protected biological structures during freezing and dehydration, restoring them intact and functional as soon as the protein was reconstituted; it also inhibited the binding of BMP-2 to Ca-P materials, which in turn lead to enhanced BMP-2 release. In the CDHA/BMP-2/SCS system, BMP-2 was lyophilized on the CDHA scaffolds without protection and then the lyophilized CDHA/BMP-2 was coated with sulfated chitosan. Even though the cumulative *in vitro* release of BMP-2 reached to over 60%, the volume percentage of new bone formation by this system was about 40% at 8 weeks post operation, which was similar to this study at only 5 weeks post operation. Therefore, both the stabilization of growth factors bioactivity and sustained release by addition of trehalose are important for the effective delivery system for bone regeneration.

### Conclusions

In summary, addition of trehalose could sufficiently preserve BMP-2 bioactivity during lyophilization and achieve enhanced and sustained BMP-2 release from lyophilized CDHA scaffold. Lyophilized BMP-2/CDHA scaffold with trehalose promoted osteogenic differentiation of bMSCs and new bone formation in critical-sized rat cranial defects when compared with lyophilized BMP-2/CDHA scaffold and absorbed BMP-2/CDHA scaffold. These results might lay a promising framework for future study by employing trehalose to preserve growth factor bioactivity and optimize the release profile of Ca-P based delivery system for enhanced bone regeneration.

## Materials and Methods

### Scaffold Preparation

Calcium phosphate cement (CPC) powder was prepared by mixing tetracalcium phosphate (TECP) and dicalcium phosphate anhydrous (DCPA) in a molar ratio of 1∶2, and the final product of hydration was CDHA with a 1.50 Ca/P ratio. CDHA scaffolds were then prepared by a particulate-leaching method. Briefly, the CPC powder was mixed with distilled water using a spatula at a powder/liquid mass ratio of 3∶1 to form a paste. Sodium chloride (NaCl) particles sieved with diameters of 400–500 µm as porogen were added into the CPC paste. The blend ratio of NaCl/CPC was 1.6∶1 (w/w). The mixture of CPC paste/NaCl was placed in a stainless steel mold (5 mm in diameter, 2 mm in height), and the mixture was molded under a pressure of 2 MPa. After storage in beakers in a constant temperature oven at 37°C and 100% relative humidity for 2 days, the samples were then immersed in deionized water to leach out the porogen. Finally, they were vacuum-dried to obtain sponge-like scaffolds. The scaffolds used for BMP-2 delivery had volume porosity of 75% with average pore diameter of 450±50 µm [44].

### BMP-2 Radioiodination and Loading

To determine the *in vitro* release profiles, the loaded BMP-2 (Shanghai Rebone Biomaterials Co., China) was radiolabeled with ^125^I according to the iodogen method [57]. Briefly, iodogen was dissolved in chloroform at a concentration of 1 mg/ml. 25 µl of this solution was used to coat a test tube and was subsequently evaporated to dryness. To this tube, 50 µl of a 1 mg/ml solution of BMP-2 and 5 µl of an 18 GBq/ml solution of Na^125^I were added to initiate the labeling. The mixed solution was incubated and stirred for 30 min at room temperature. Sephadex column chromatography was then applied to separate free iodine from the radioactive product. After elution with 0.01 M PBS (pH 7.4) at a flow rate of 1 ml/min for 5 min, the ^125^I-BMP-2 was collected. Trichloroacetic acid (TCA) precipitation of the final ^125^I–BMP-2 solution indicated 99% precipitable counts. The final ^125^I-BMP-2 concentration was adjusted to 0.2 mg/ml in PBS.

For the lyophilization group, the ^125^I-BMP-2 solution with 0.3 M trehalose [17] (lyo-tre-BMP-2) or without trehalose (lyo-BMP-2) were dripped onto CDHA scaffolds at a fixed volume of 30 µl per sample. In this way, each sample was loaded with 6 µg ^125^I-BMP-2. The samples were pre-freezed at -80°C for 3 hours and then further lyophilized in a freeze-drier (Labconco FreeZone, Labconco, Kansas City, MO) for 24 hours. For the absorption BMP-2 group, the same BMP-2 solution with 0.3 M trehalose (abs-tre-BMP-2) or without trehalose (abs-BMP-2) was physically dripped onto the scaffolds for 30 minutes before use.

### Ethics Statement

The Ethics Committee for Animal Research at the Ninth People’s Hospital affiliated to Shanghai Jiao Tong University approved all the experimental protocols involving the use of rats.

### Culture of Rat bMSCs

Six-week-old male Fisher 344 rats were obtained from the Ninth People’s Hospital Animal Center (Shanghai, China). Rat bMSCs were isolated and cultured as previously described [58]. Cells were cultured in Dulbecco’s modified Eagle’s medium (DMEM) (Gibco BRL, Grand Island, NY, USA) with 10% FBS (Hyclone, Logan, UT, USA), 100 units/ml penicillin and streptomycin at 37°C in an atmosphere of 5% CO_2_. The cells used for this study required 3 passages each, with about 2 doublings per subculture stage.

### *In vitro* BMP-2 Release and Bioactivity

To determine the release kinetics of BMP-2 from CDHA scaffolds, the samples were incubated in 1 ml PBS. The elution of BMP-2 was examined at 1 hour and 1, 3, 7, 10, 14, 21, 28 days. At each time point, the buffer was removed and replaced with fresh PBS. Release was quantified by monitoring the radioactivity in the removed buffer using a gamma counter (Shanghai Institute of Nuclear Instrument Factory, China). All ^125^I counts were corrected for decay and normalized to the starting amount.

To determine the bioactivity of BMP-2 released from the scaffolds, releasates from the first 7 days from each sample separately were combined and examined for alkaline phosphatase (ALP) activity using bMSCs. Cells were seeded on 48-well plates at a concentration of 2×10^4^ per well. The pooled BMP-2 releasates of different formulations were adjusted to 100 ng/ml based on the cumulative BMP-2 release of each group. 24 hours after seeding, the culture medium was changed to DMEM containing 5% FBS with or without 100 ng/ml of BMP-2 releasate (0.5 ml). The same amount of fresh BMP-2 was used as a positive control. The cell culture medium was changed every two days. At days 3, 7 and 14, cells were lysed with 80 µl of 0.05% Triton X-100. The lysates (20 µl) were added to 100 µl of substrate buffer (2 mg/ml disodium p-nitrophenylphosphate hexahydrate and 0.75 M 2-amino-2-methyl-1-propanol). After incubation of the mixtures at 37°C for 30 min, absorbance at 405 nm was measured. The ALP activity was normalized by the total protein content determined using the bicinchoninic acid (BCA) assay (Pierce Biotechnologies, Rockford, IL) [59].

### Effect of BMP-2-loaded CDHA Scaffolds on bMSCs Proliferation

Proliferation of bMSCs cocultured with BMP-2/CDHA scaffolds was determined by a 6-well Transwell culture system (0.4 mm, Corning, NY, USA). BMSCs were concentrated to 5×10^4^ cells/ml (200 µl) and then plated into the lower well. The scaffolds were placed in the upper well and the medium was changed every 2 days. On days 1, 3, 7, and 14, bMSCs were collected to quantify DNA content by use of Hoechst 33258 (Sigma, USA) as previously described [60]. Proliferations of cells cocultured with CDHA alone scaffold and no graft were also detected.

### Effect of BMP-2-loaded CDHA Scaffolds on bMSCs Osteogenic Differentiation

Osteogenic differentiation of bMSCs cocultured with BMP-2/CDHA scaffolds by the same Transwell culture system was detected on days 3, 7 and 14. The total RNA was extracted with TRIzol Plus RNA purification kit (Invitrogen, USA) according to the manufacturer’s instructions. Highly purified gene-specific primers for run×2 (Run×2), osteopontin (OPN), osteocalcin (OCN), and bone sialoprotein (BSP) and the calibrator reference gene, GAPDH, were synthesized commercially (Shengong, Co. Ltd. Shanghai, China), and the specific primers sets are outlined in Table S1. All RT-qPCRs of bone marker genes were carried out six times with a 7900HT sequence detection system (Applied Biosystems). Analysis was based on calculating the relative expression level of the bone marker genes compared to the expression of bMSCs with no graft on days 3, 7 and 14.

The protein levels of OPN and OCN secreted from bMSCs cocultured with different formulations were measured at days 3, 7 and 14. The rat commercial ELISA kits (antibodies-online Inc., Atlanta, GA, USA) were used to determine protein contents in cell culture supernatant according to the manufacturer’s instructions. Analysis of bMSCs cocultured with CDHA alone scaffold was treated as controls. All experiments were done in triplicate.

### Preservation of Protein Bioactivity for Lyophilized Formulation

Lyophilized BMP-2/CDHA samples with addition of trehalose were stored at different temperature conditions: −20°C, 4°C, and 25°C. Each sample was completely sealed in the microcentrifuge tubes to inhibit humidification. After 2 and 5 weeks storage, the samples were incubated in PBS and 100 ng/ml of BMP-2 releasate from the first 7 days was collected for ALP activity assay to determine the bioactivity of BMP-2 released from the scaffolds. At days 7, the ALP activity was assessed according the method mentioned above. Fresh BMP-2 without lyophilization was used as a standard for the ALP assay to determine the change of protein bioactivity with different storage time and temperature.

### Preparation of Cell-material Composite

For the preparation of bMSCs/BMP-2/CDHA constructs, 20 µl of bMSCs solution were seeded onto BMP-2 loaded CDHA scaffolds and CDHA alone scaffolds at a concentration of 2×10^7^ cells/ml for 4 hours before implantation. In a parallel experiment, the scaffolds were prepared and seeded with bMSCs at an identical cell density. After 24 h and 7 days incubation, the constructs were fixed in 2% glutaric dialdehyde for 2 hours, cut into two halves and then characterized by scanning electron microscopy (SEM) (Philips Quanta-200, Amsterdam, Netherlands).

### Surgical Procedure and Step-wise Fluorescent Labeling

A total of 45 male Fisher 344 rats, aged 12 weeks old, each with a weight of 250 g ±15 g were enrolled in the study. The animals were anaesthetized by intraperitoneal injection of pentobarbital sodium (40 mg/kg). For local anesthesia, 0.5 ml of 1% lidocaine with epinephrine (1∶100,000) was injected subcutaneously. The dorsal part of the cranium was shaved, aseptically prepared for surgery, and a sagittal incision of approximately 20 mm opened over the scalp of the animal. The periosteum was removed and a full-thickness bone defect (5 mm in diameter) was trephined in the centre of each parietal bone using a slow speed dental drill that was cooled continuously by 0.9% saline solution irrigation. The calvarial defects were randomly repaired by the following implants: (1) lyo-tre-BMP-2 CDHA scaffolds with bMSCs (*n* = 18); (2) lyo-BMP-2 CDHA scaffolds with bMSCs (*n* = 18); (3) abs-BMP-2 CDHA scaffolds with bMSCs (*n* = 18); (4) CDHA scaffolds with bMSCs (*n* = 18) and (5) CDHA alone (*n* = 18). The wound was closed in layers using 4–0 resorbable sutures. Animals were given 300,000 units penicillin G per day for 3 days after surgery to prevent infection.

At 2 and 4 weeks after the operation, the animals were intraperitoneally administered with 30 mg/kg alizarin red S (AL, Sigma, USA), and 20 mg/kg calcein (CA, Sigma, USA), respectively.

### *In vivo* BMP-2 Release Measurement

To determine the release kinetics of BMP-2 *in vivo*, an unilateral rat calvarial defect model was employed to avoid interference by radioactive source from the other side of the calvarial defects. Another 18 male Fisher 344 rats were enrolled in the assay. The unilateral defect was made in the left side of each parietal bone and implanted with (1) lyo-tre-BMP-2 CDHA scaffolds (*n* = 6); (2) lyo-BMP-2 CDHA scaffolds (*n* = 6); (3) abs-BMP-2 CDHA scaffolds (*n* = 6). The release kinetics from the implanted scaffolds was followed up by a non-invasive method as previously described [61]. This method permitted periodic assessment of the remaining ^125^I-BMP-2 at the defect site using an external probe-type gamma counter (CaptusVR, Nuclear Iberica). Briefly, at each sampling time point, five 1-min readings were taken at the ^125^I emission peak (maximum 27 KeV) and the mean was accepted as the remaining radioactivity. The ^125^I counts were corrected for decay and normalized to the postoperative measurement at day 0.

### μ-CT Scan

Five weeks post-operation, animals were euthanized by intraperitoneal overdose injection of pentobarbital sodium (100 mg/kg). The specimens, containing the 5 mm cranial defect and 3–5 mm peripheral cortical bone adjacent to the defect, were fixed in 4% phosphate-buffered formalin solution. Bone blocks (*n* = 6 for each group) were examined on a micro-CT system (µCT-80, Scanco Medical AG, Bassersdorf, Switzerland). The specimens were placed in a sample holder filled with water. They were oriented in such a way that the plan of the block was parallel to the axis of the sample holder. A high-resolution protocol (pixel matrix, 1024*1024; voxel size, 36 µm; slice thickness, 36 µm) was applied. Depending on the length of the specimens, up to 200 slices were scanned perpendicular to the block. To determine the amount of newly formed bone tissue, the best threshold for the CDHA scaffold alone was selected, followed by determination of the threshold for the CDHA scaffold and newly formed bone together. In addition, the ranges and means of the gray levels characteristic of the CDHA scaffold and newly formed bone were determined. The CDHA scaffold showed a mean gray level of 150±20, while that of the newly formed bone was 55±15. The determined threshold to separate CDHA scaffold from newly formed bone was set at 100, allowing reliable distinction between the two tissue types. Finally, µCT slices were compared with the corresponding histological slides to verify the reliability of the discrimination criteria [31], [53], [54]. The mean volume of three unimplanted CDHA scaffolds was assessed with µCT as reference to calculate both the volume percentages of new bone formation and remnant scaffold within the defect site. The amount of new bone formation was calculated by dividing newly formed bone voxels by mean CDHA scaffold volume. Resorption was calculated by dividing the voxels of the ceramic that was present 5 weeks post-operation by the voxels of the mean CDHA volume. Three-dimensional images were reconstructed and the data was calculated using its auxiliary software (Scanco Medical AG).

### Histology and Histomorphometry Analysis

After μ-CT analysis, samples from each group were used for histological observation. For processing undecalcified sections, specimens (*n* = 6 for each group) were dehydrated in ascending concentrations of alcohols from 75% to 100%, and finally embedded in polymethymethacrylate (PMMA). Longitudinal sections were cut to 150 µm thick using a microtome (Leica, Hamburg, Germany), and were subsequently ground and polished to a final thickness of about 40 µm. Undecalcified sections were observed for fluorescent labeling using a microscope under confocal laser scanning microscope (CLSM) (Leica TCS Sp2 AOBS, Germany). Excitation and emission wavelengths of chelating fluorochromes were used 543/617 nm and 488/517 nm for alizarin red S (red) and calcein (green), respectively. The fluorochrome staining of new bone was quantified at four locations that equally divided the defect site between the two ends of host bone along the longitudinal sections. Four photographs were taken from the same area for each of the four locations: Two fluorescence microscopy images including the fluorochromes alizarin red S and calcein, and one merging image of two fluorescent labels, and one regular image under transmission light microscopy without a specific filter combined with the former merged image. The microscope images were then evaluated using a picture-analysis system (Image-Pro PlusTM, Media Cybernetic, Silver Springs, MD, USA). The number of pixels labeled with each fluorochrome in each image was determined as a percentage of the mineralization area. The mean value of the four measurements was calculated per section, three randomly selected sections from the serial sections collected from each sample were analyzed, and then they were used to calculate average values for each group (*n* = 6). This quantitative analysis was done separately for red (alizarin red S) and green (calcein) which represent new bone regeneration and mineralization 2 and 4 weeks post-operation.

In the merging image of two fluorescent labels which was acquired as described above, the mineral apposition rate was calculated by the vertical distance between the two fluorescent labels divided to 14 days. The mean value of the four locations was calculated per section, three randomly selected sections from the serial sections collected from each sample were analyzed, and then they were used to calculate average values for each group quantitatively (*n* = 6).

After observation for area of fluorochromed stained bone and mineral apposition rate, the undecalcified sections were stained with Van Gieson’s picro fuchsin for quantitative assessment of the percentage of new bone area and remnant scaffold area. The percentages of newly formed bone area was calculated by new bone area divided to the total final repaired area which included the repaired area between the two ends of host bone (5 mm in length) along the longitudinal sections at low magnification (12.5×). The measurements on undecalcified specimens were performed using the same picture-analysis system. Three randomly selected sections from the serial sections collected from each sample were analyzed, and they were used to calculate average values for each group (*n* = 6). The percentage of remnant scaffold area was calculated by the same method.

For processing decalcified paraffin sections, specimens (*n* = 6 for each group) were decalcified, embedded in paraffin and sectioned longitudinally into 4 µm thick sections for qualitative assessment of newly formed bone tissue by HE staining (to observe tissue and cell morphologies), Goldner’s trichrome staining (to observe the condition of osteogenesis and mineralization) and Masson’s trichrome staining (to observe maturation degree of collagen). The tissue in the repaired area between the two ends of host bone was assessed using a light microscope at high magnification (200×, Carl Zeiss, Inc. Germany).

### Biomechanical Testing

After sacrifice, six specimens were used for mechanical testing for each group. Non-destructive micro-compression on the calvaria defects was performed using a universal testing machine (AG-2000A, Shimadzu, Japan) at a loading rate of 1 mm min^−1^. An indenter probe of 0.5 mm diameter was micro-fabricated for the test. Micro-compressions of up to 50% strain were conducted at an average of six different locations on each defect site, and the load-displacement and stiffness (compression modulus) were determined. The probe locations were uniformly distributed on the surface of the constructs.

Push-out tests were conducted to evaluate the functional mechanical integration of the tissue-engineered constructs into the host calvaria, and were performed on the same testing machine. An indenter probe of 4.5 mm diameter, slightly smaller than the scaffold diameter of 5 mm, was fabricated for the test. After the micro-compression tests were completed, the calvarial specimens were tested to failure and the final yield was determined. To determine the micro-compression and push-out strength of normal rat calvaria and compare them with different constructs, six normal calvaria samples from three extra animals with the same condition were enrolled.

### Statistical Analysis

Statistically significant differences between the various groups were measured using one way ANOVA and Student-Newman-Keuls (SNK) post hoc. All statistical analysis was carried out using an SAS 6.12 statistical software package (SAS, Cary, NC, USA). All the data are expressed as mean ± standard deviation. P values <0.05 were considered significant.

## Supporting Information

Figure S1 **SEM images of the surface** **of different scaffolds and constructs.** The surface morphology of lyo-tre-BMP-2 CDHA scaffold (A1), lyo-BMP-2 CDHA scaffold (B1), abs-BMP-2 CDHA scaffold (C1) and CDHA scaffold (D1). After 1 day combination, cells were fully spreading and growing (A2–D2). Nominal differences in cellular adhesion were observed among each group. At days 7 after cell seeding, the pores of lyo-tre-BMP-2 CDHA scaffold were deposited with abundant, dense extracellular matrix associated with cell layers (A3). Visually, less extracellular matrix formed on the lyo-BMP-2 and abs-BMP-2 CDHA scaffolds (B3, C3) than that on the lyo-tre-BMP-2 scaffolds. Only a thin cell layer grew on the surface of CDHA scaffold (D3). Scale bar = 5, 25 and 150 µm for A1–D1, A2–D2, and A3–D3 respectively.
(TIF)

Table S1 **Primers for real-time PCR.**
(DOC)
