# Supplementary figures and images for: Trehalose Maintains Bioactivity and Promotes Sustained Release of BMP-2 from Lyophilized CDHA Scaffolds for Enhanced Osteogenesis In Vitro and In Vivo
Source: PLoS One. 2013 Jan 24;8(1):e54645. doi: 10.1371/journal.pone.0054645 (PMC3554655; doi:10.1371/journal.pone.0054645)

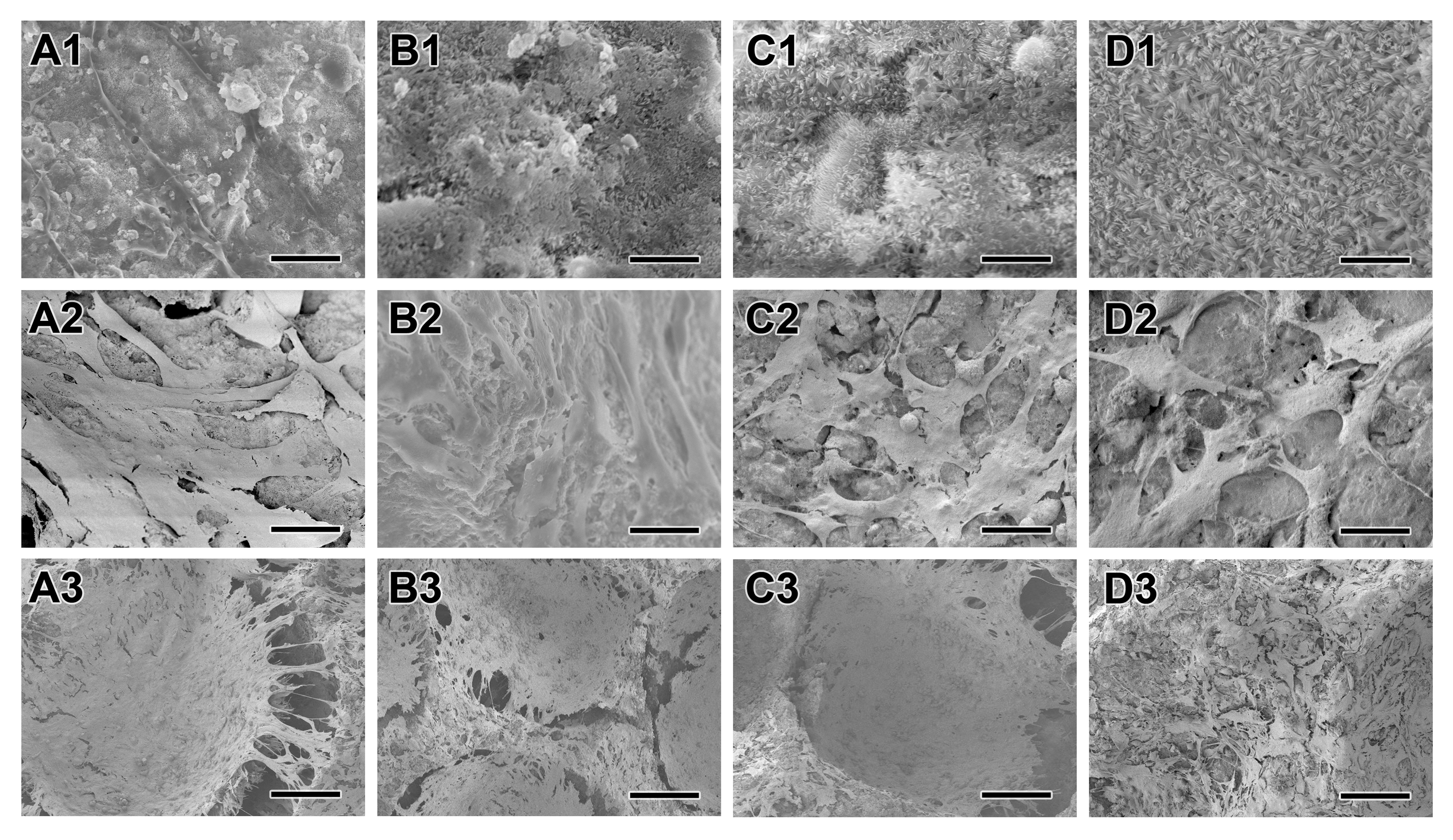

Supplement: Figure S1 — SEM images of the surface of different scaffolds and constructs. The surface morphology of lyo-tre-BMP-2 CDHA scaffold (A1), lyo-BMP-2 CDHA scaffold (B1), abs-BMP-2 CDHA scaffold (C1) and CDHA scaffold (D1). After 1 day combination, cells were fully spreading and growing (A2–D2). Nominal differences in cellular adhesion were observed among each group. At days 7 after cell seeding, the pores of lyo-tre-BMP-2 CDHA scaffold were deposited with abundant, dense extracellular matrix associated with cell layers (A3). Visually, less extracellular matrix formed on the lyo-BMP-2 and abs-BMP-2 CDHA scaffolds (B3, C3) than that on the lyo-tre-BMP-2 scaffolds. Only a thin cell layer grew on the surface of CDHA scaffold (D3). Scale bar = 5, 25 and 150 µm for A1–D1, A2–D2, and A3–D3 respectively. (TIF) [file pone.0054645.s001.tif]
