# Supplementary material for: Trehalose Maintains Bioactivity and Promotes Sustained Release of BMP-2 from Lyophilized CDHA Scaffolds for Enhanced Osteogenesis In Vitro and In Vivo
Source: PLoS One. 2013 Jan 24;8(1):e54645. doi: 10.1371/journal.pone.0054645 (PMC3554655; doi:10.1371/journal.pone.0054645)
Supplement: Table S1 — Primers for real-time PCR. (DOC) [file pone.0054645.s002.doc]

**Table S1 Nucleotide sequences for realtime RT-PCR primers**

| Genes | Primer sequence (forward/reverse) | Product size (bp) | Annealing temperature(°C) | Accession number |
| --- | --- | --- | --- | --- |
| GAPDH | 5’-CCTGCACCACCAACTGCTTA-3’/5’-GGCCATCCACAGTCTTCTGAG-3’ | 140 | 58 | NM_017008 |
| Runx2 | 5’-GCTTCTCCAACCCACGAATG-3’/5’-GAACTGATAGGACGCTGACGA-3’ | 212 | 58 | XM_346016 |
| OPN | 5’-GACGGCCGAGGTGATAGCTT-3’/5’-CATGGCTGGTCTTCCCGTTGC-3’ | 208 | 58 | NM_012881 |
| OCN | 5’-AAAGCCCAGCGACTCT-3’/5’-CTAAACGGTGGTGCCATAGAT-3’ | 232 | 58 | NM_013414 |
| BSP | 5’-GATAGTTCGGAGGAGGAGGG-3’/5’-CTAACTCCAACTTTCCAGCGT-3’ | 172 | 58 | NM_012587 |
